# Outcomes following calcium channel blocker exposures reported to a poison information center

**DOI:** 10.1186/s40360-018-0271-9

**Published:** 2018-11-27

**Authors:** Mikkel B. Christensen, Kasper M. Petersen, Søren Bøgevig, Salam Al-Gibouri, Espen Jimenez-Solem, Kim P. Dalhoff, Tonny S. Petersen, Jon T. Andersen

**Affiliations:** 1 Department of Clinical Pharmacology, Copenhagen University Hospital Bispebjerg, Bispebjerg Bakke 23, DK-2400 Copenhagen, Denmark; 2 Faculty of Health and Medical Sciences, University of Copenhagen, Copenhagen, Denmark

**Keywords:** Overdose, Poisoning, Calcium channel blockers, Calcium antagonist, Verapamil, Amlodipin, Felodipin, Isradipin, Lacidipin, Lercanidipin, Nifedipin, Nimodipin, Nitrendipin, Diltiazem

## Abstract

**Background:**

Calcium channel blockers (CCBs) are widely used drugs that have a narrow therapeutic index. Even minor overdoses must be treated in-hospital due to the risk of severe hypotension and bradycardia. We aimed to describe trends in CCB use and overdoses in Denmark.

**Methods:**

Data on enquiries concerning CCBs reported to the Danish Poisons Information Center (DPIC) from January 2009 to January 2015 was coupled with data on hospitalization and mortality obtained from Danish National Registers. We obtained data on the general use of CCBs in Denmark and retrieved medical charts on fatal cases.

**Results:**

From a total of 126,987 enquiries to the DPIC in 2009–2014 we identified 339 CCB unique exposures (3‰ of all). Children < 5 years accounted for 20% all exposures and these were classified as ‘intake during playing’ (61%) and ‘medication errors’ (39%). Among adults ‘suicidal poisonings’ (58%), and ‘medication errors’ (34%) were most frequent. A majority (81%) of exposures led to hospital admission. Seven patients (2%) died from the CCB exposure and all were adults with ‘suicidal poisoning’. Amlodipine accounted for 95% of all CCB prescriptions, was involved in 71% of enquiries and in 29% of fatalities. Verapamil accounted for 3% of prescriptions, was involved in 13% of enquiries and 57% of fatalities.

**Conclusion:**

Four fifths of enquiries to the DPIC result in hospitalization and one fifth concern small children. Mortality were infrequent and occurred only in adults with suicidal exposures and with and an overrepresentation of verapamil exposures.

## Background

Calcium channel blockers (CCBs) are widely used drugs indicated for the treatment of cardiovascular disease and migraine. CCBs have a narrow therapeutic index, and consequently even minor overdoses have to be treated in-hospital due to the risk of severe hypotension and bradycardia [1, 2]. All of the CCBs block L-type voltage gated calcium channels, but individual agents differ in chemical structure and tissue selectivity [3]. At therapeutic doses CCBs belonging to the dihydropyridine class (e.g. amlodipine and felodipine) are primarily affecting calcium channels in the smooth muscle in peripheral vessels, whereas non-dihydropyridine agents (verapamil and diltiazem) are also affecting calcium channels in the heart [2, 3]. Tissue selectively has been reported to be attenuated with increasing doses, but nonetheless, non-dihydropyridine overdoses often leads to various degrees of conduction block and are therefore considered most dangerous [1, 2]. Metabolic and central nervous system (CNS) disturbances are also seen after CCB overdoses, but the cause of death after CCB toxicity is usually presumed to be circulatory collapse [1–4]. Thus, advising about and caring for patients, who have ingested CCBs deliberately or unintentionally is a challenging health care task; and there is limited information on the pharmacoepidemiology and outcomes of overdoses with CCBs. The objectives of this study are to describe trends in the general use of CCBs and to describe causes and consequences of CCB poisonings based on data from the Danish Poisons Information Center (DPIC), Danish national registers and medical charts.

## Methods

### Data

In this retrospective study we identified all patients poisoned with CCBs from the DPIC-database from January 1st, 2009 to December 31st 2014. The DPIC is a telephone-based, 24-h service providing information on a national level to guide the public and health care professionals on all aspects related to acute poisonings including the management of the poisoned patient. All telephone enquiries to the DPIC are registered in a database with information on the suspected poisoning. The following are recorded: patient data including the unique personal identification number, a description of the poisoning (poison, amount in DDD, mode of exposure, etc.), clinical status of the patient, and the cause of poisoning. The cause of poisoning was divided into ‘suicidal intake’ or ‘accidental intake’, ‘abuse’ or ‘other’. Accidental intake includes ‘intake during playing’ and ‘medication errors’ e.g. incorrect dosage or drug, accident, or confusion of pills.

In the DPIC-database we identified all inquiries concerning CCBs by searching for synonyms of CCBs combined with all generic and brand names of CCBs marketed in Denmark during the study period. Only records with a complete personal identification number and a registration of a possible CCB overdose or poisoning were included in the study. The DPIC records of CCB exposures were then linked with hospital records from the Danish National Hospital Register [5] and information on death was retrieved from the Danish Register of Causes of Death [6]. All records were linked using the unique Danish personal identification number [7], which all persons living in Denmark get at birth or following immigration to Denmark.

The National Hospital Registry contains information on all hospitalizations and outpatient visits in Denmark, including 99% of all discharge records, length of hospital stay and discharge diagnoses (International Classification of Diseases 10th Edition (ICD-10) Danish revision) [5]. The Danish Register of Causes of Death holds information on all deaths in Denmark including place, time, and cause of death classified using ICD-10 Danish revision codes [6].

Sales numbers for CCBs expressed in Defined Daily Doses (DDDs) in the primary and secondary sectors in Denmark were extracted as additional information from the online database MEDSTAT [8].

Detailed medical charts for the fatal cases (identified through national registers) were retrieved from the hospital departments, where these patients were admitted.

### Statistics

All analyses and data management were performed using SAS statistical software version 9.4 (SAS Institute Inc., Cary, NC, USA). Frequency distributions comparisons were analysed with *Fisher’s exact test*. For all analyses, a two-sided value of *p* < 0.05 was considered statistically significant, and all odds ratios are presented with 95% confidence intervals. Graphical presentation was prepared using Graphpad Prism version 7.02 (La Jolla, CA, USA).

## Results

**Fig. 1:**
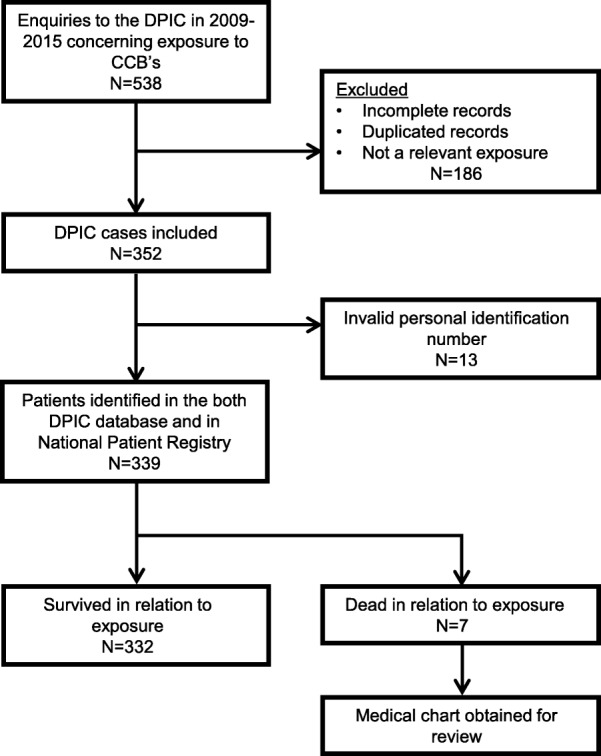
Flow Chart for patient inclusion

From a total of 126,987 enquiries to the DPIC in 2009–2014 we identified 339 records (3‰ of all) concerning patients with a CCB exposure, where outcome data was available through the national registries (Fig. 1).

### Description of population

#### Age and gender distribution

**Fig. 2:**
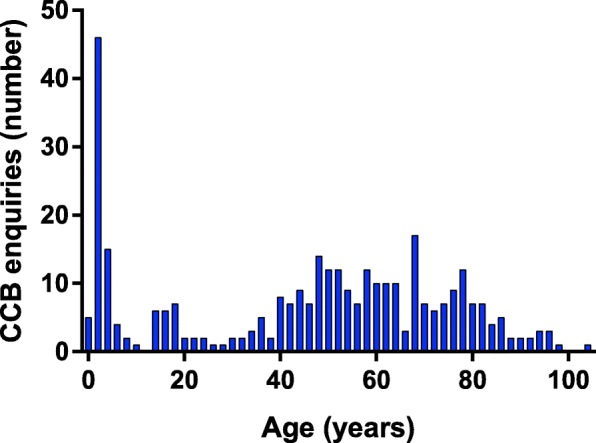
Age distribution among CCB exposures registered by the Danish Poison Information Center from 2009 to 2014

The age distribution among patients with a CCB exposure was bimodal with a peak in the preschool children and in adults aged 40–80 years (Fig. 2).

Children (i.e. boys and girls < 16 years) accounted for 24% of all CCB enquires (*N* = 78), and the youngest age group 0–5 years accounted for 20% all exposures (*N* = 69). There were slightly more boys (56%) among exposed children, whereas females were the majority in individuals above 16 years of age (61%) (*p* < 0.01).

#### Reason for exposure

Suicidal exposures accounted for 46% of all enquiries. However, the reason for exposure differed across age groups. Only 6% (*N* = 5) of the children’s cases were caused by suicidal exposures, all concerning girls aged 13–15 years. The major reason for exposure (85%) in children were ‘medication errors’ (40%) or ‘intake during playing’ (54%). In the youngest age (i.e. < 5 years) group reasons for exposure were ‘intake during playing’ (61%) and ‘medication errors’ (39%). The age group 16–65 years had a high proportion (71%) of suicidal exposures compared to the age group above 65 years (35%) (*p* < 0.0001). The accidental exposures in the above 65 years age-group were most often (55%) ‘medication errors’.

#### CCBs involved in poisonings and their sales in Denmark

Single drug exposures constituted 35% of all cases, but 65% of exposures in children. The majority (78%) of accidental exposures concerned intake of only one CCB, whereas the majority (84%) of suicidal exposures the CCBs were taken with other substances (i.e. mixed exposures). Among all enquiries (i.e. both suicidal and accidental) concerning CCBs, amlodipine was the most common and was involved in 72% (*N* = 249) of the cases, whereas verapamil was involved in 13% (*N* = 45), felodipine in 5% (*N* = 16), and diltiazem in 5% (*N* = 16).

**Fig. 3:**
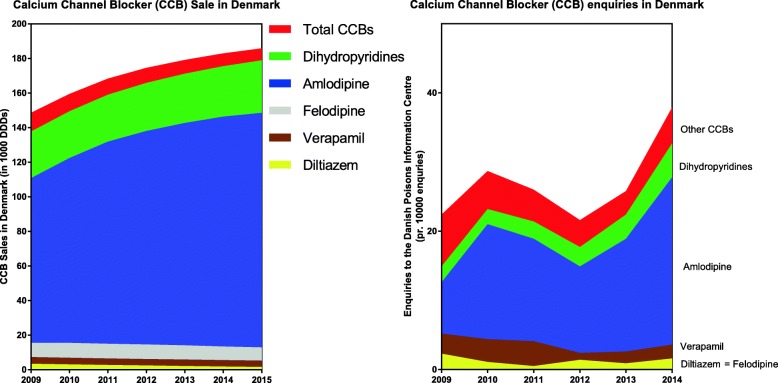
**a** Sales in WHO defined daily doses (DDD) of calcium Channel Blockers in Denmark and **b**) CCB exposures registered by the Danish Poison Information Center from 2009 to 2014

From 2009 to 2015, the sale of dihydropyridine CCBs increased, whereas sales of verapamil and diltiazem slightly decreased (Fig. 3a). The population-corrected frequency of enquiries concerning CCBs were 61 enquiries per million citizens (Fig. 3, Table 1 in appendix), with an increasing trend in enquiries over the study period (Fig. 3b).

### Outcome of exposure

#### Hospital admission

**Fig. 4:**
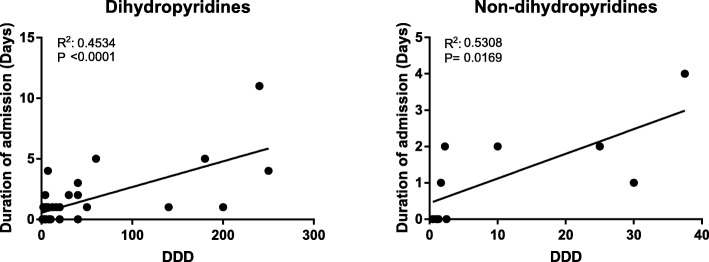
Association of dose of CCB exposure (expressed in DDD) with duration of in-hospital stay in adults for **a**) the dihydropyridines (mostly amlodipine) or **b**) non-dihydropyridines (verapamil)

A majority (81%) of cases were admitted to a hospital. Children were admitted in 88% of exposures, and adults were admitted in 78% of exposures. In adults with clear anamnestic information on exposure dose the length of in-hospital stay increased with higher DDD-intake/exposure of both dihydropyridine and non-dihydropyridine CCBs (Fig. 4). In children, the anamnestic information on dosage was too uncertain (e.g. worst-case scenarios) for the purpose of relating DDDs to length of hospital stay. In children the length of in-hospital stay had a median value of 1 day (range 0–4 days): Dihydropyridines accounted for 25 exposures in children that subdivided into: 4 exposures leading to: 0 days in-hospital stay; 17 exposures: 1 day; 3 exposures: 2 days; 1 exposure: 4 days. Verapamil intake by children accounted for six exposures: 2 exposures led to 1 day in-hospital stay and 4 exposures led to 3 days in-hospital stay.

#### Mortality following CCB exposure

Seven patients (2%) died within 30 days after CCB exposure, but one of the patients did not die from CCB poisoning (Table 2 in Appendix presents details from these lethal cases). Mortality occurred only in adults with suicidal exposures, and the lethal cases were all mixed exposures. The CCB’s involved were verapamil (*N* = 4), amlodipine (*N* = 2) or felodipine (*N* = 1). Of the four adults who died after verapamil exposure, information on intake was available for two, who ingested 25 and 100 DDDs, respectively. There were no deaths among 120 enquiries concerning an isolated intake of dihydropyridine CCB (both adults and children) – even after a severe overdose (e.g. in 6 patients with exposures exceeding 100 DDDs).

## Discussion

We report that despite enquiries to the DPIC concerning CCBs being relatively infrequent, four-fifth of exposures resulted in hospitalization and 2% led to death. In that context, we find it reassuring that mortality only occurred in adults with suicidal intent. One fourth of exposures concerned children, and there were no deaths or prolonged in-hospital stays in pre-school children.

Our data may therefore be used to question the relatively cautious guidelines for out-of-hospital management of CCB overdoses recommending that intake of amlodipine and felodipine doses of 0.3 mg/kg and 10 mg (2 DDDs) in children and adults, respectively, should be referred to the emergency department [1]. Our data is quite accurate on length of hospital stay and there were only very few in-hospital stays > 1 day in children after accidental dihydropyridine or verapamil overdoses. A similar finding was also reported by others, where intake of a relatively large amount of CCBs did not give rise to major symptoms in children under the age of 6 years [9, 10]. However, our data material is not precise enough on symptomatology or large enough to contradict reports of significant hypotension or severe toxicity even after minor accidental exposures ( [1, 11].

The finding of no fatalities in adults following accidental exposures (i.e. not counting suicidal or abuse exposures), are in line with other reports. Generally deaths have only rarely been reported following unintentional intake of CCBs and often other important complicating factors may have played important roles for the fatal course e.g. serious co-exposures such as other cardioactive drugs, co-morbidity such as heart failure, or very low age [1, 12]. It is noteworthy that there were no deaths in adults following isolated intake of dihydropyridines, which are by far the most widely used CCBs, even after intake of up to 200 DDDs. In contrast, verapamil was involved in four out of seven deaths despite only being involved in 13% of enquiries and in 3–5% of the total CCB sale in Denmark. Thus, our results seem to corroborate the larger risk attributed to verapamil also reported by others [1, 4, 13]. Our thorough review of the fatal cases underlines the differences between verapamil and dihydropyridines in toxicity: All verapamil overdoses presented with severe cardiotoxicity and conduction abnormalities (i.e. 3rd degree atrioventricular- and/or left bundle branch blocks, pulseless electric activity) that deteriorated after arrival and led to death within 3 days (Table 2 in Appendix). Of the two fatal dihydropyridine cases (one amlodipine and one felodipine), the felodipine exposure did not present with or develop in-hospital severe cardiac disturbances but died after 8 days of hypoxic-ischemic brain damage (developed before arrival to the hospital and likely related to co-ingestion of codeine and quinine). The fatal amlodipine exposure died of severe cardiotoxicity but had also co-ingested metoprolol and enalapril – whereof both, but in particular metoprolol is known to exert synergistic cardiodepressive effects with CCBs.

A particular problem evident from the fatal cases presented in Table 2 in Appendix is the apparent lack of alignment with current treatment recommendations [14], which in all cases was advocated by the DPIC. Particular, therapies such as high-dose insulin (used in two of six cases), high-dose glucagon (used in three of six cases), and high-dose intravenous lipid emulsion (used in one of six cases), seem underused. Findings of lack of adherence to guidelines and poison center advice have also been reported by others [15, 16].

Collectively, our findings correlate reasonably with other publications describing CCB exposures [1, 9, 17–20]. However, there are some discrepancies worth noticing. Our rate of hospital admission (81%) appears high compared to previous reports of approximately 50–73% hospital admissions following CCB exposures [1, 17] and may reflect the severity of exposures reported to the DPIC, where approximately half of the exposures were suicidal. Olson et al. [1] described death to occur only after verapamil, nifedipine, and diltiazem and Deters et al. [18] described death to occur most frequently after diltiazem exposures. In our material, nifedipine and diltiazem was only involved in few exposures and not involved in fatal cases. This difference likely reflects the relatively infrequent use of nifedipine and diltiazem in Denmark, where sales are decreasing for older dihydropyridines (e.g. nifedipine) and non-dihydropyridines (verapamil and diltiazem).

### Strengths and limitations

This study covers nationwide follow-up data in our study population due to the completeness of the Danish National Health Registers. Thus, we have reliable outcome data on all patients studied. Nevertheless, the major limitations of this study relate to the completeness of data: We identified exposures through the enquiries to the DPIC, and therefore these cases may not be representative all the CCB exposures in Denmark; some exposures may be handled at home or be hospitalized or die without contact to the DPIC. Furthermore, as discussed previously we have limited data on clinical symptoms and our data reflects the clinical scenario in acute poisonings, thus anamnestic information concerning exposures, i.e. dose and potential co-ingestants are somewhat uncertain – and in this retrospective study no formal validation (i.e. measurement of plasma concentration etc.) of offending drugs could be performed.

## Conclusion

From 2009 to 2014, the sale of dihydropyridine CCBs increased in Denmark, whereas sales of verapamil and diltiazem slightly decreased. In the same period, enquiries to the DPIC concerning CCBs were rare (3‰ of all enquiries), but often serious and with 80% resulting in hospitalization. One fourth of exposures concerned children and most of these were monoexposures. There were no deaths among 120 enquiries concerning an isolated intake of dihydropyridine CCB (both adults and children) – even after a severe overdose (e.g. in 6 patients with exposures exceeding 100 DDDs). Amlodipine accounted for 95% of all CCB prescriptions, was involved in 71% of enquiries and in 29% of fatalities. Verapamil accounted for 3% of prescriptions, was involved in 13% of enquiries and 57% of fatalities. Mortality occurred in 2% (*N* = 7) and only in adults with suicidal exposures.

## Appendix

**Table 1:** Citizens, CCB-related enquiries, and CCB sales and in Denmark 2009–2014

Year	2009	2010	2011	2012	2013	2014
Danish population in persons	5,532,531	5,557,709	5,579,204	5,599,665	5,623,501	5,655,750
CCB related enquiries	40	52	57	44	61	89
Total CCB sale in DDD	148,626	159,483	168,374	174,585	179,149	182,916
Dihydropyridines sale in DDD (% of total CCB sale)	137,779 (93)	149,428 (94)	159,057 (94)	165,904 (95)	171,181 (96)	175,518 (96)
Amlodipine sale in DDD (% of total CCB sale)	110,792 (75)	122,425 (77)	131,922 (78)	138,074 (79)	142,678 (80)	146,330 (80)
Verapamil sale in DDD (% of total CCB sale)	7314 (4.9)	6940 (4.4)	6543 (3.9)	6201 (3.6)	5872 (3.3)	5566 (3.0)
Diltiazem sale in DDD (% of total CCB sale)	3409 (2.3)	3079 (1.9)	2764 (1.6)	2480 (1.4)	2096 (1.2)	1832 (1.0)

**Table 2:** Fatal cases

Gender and age	Comorbidity	Anamnestic drug exposure *(doses noted if known)*	Interval to presentation and death post intake	Clinical presentation at hospital *Time from intake to admission if known) and symptoms (GCS, temperature, hemodynamic, renal, other*	Paraclinical parameters *Arterial blood gas (pH, base excess, lactate) blood samples (*e.g. *glucose, calcium,) and ECGs*	Treatment *Gastrointestinal decontamination (*e.g. *gastric emptying, activated charcoal).* *Inotropes, vasopressors and vasoactive therapy (*e.g. *isoprenaline, norepinephrine).* *Other interventions (*e.g. *dialysis, calcium, glucagon)*
Female, 82 y	Morbus Meniere. Cardiovascular disease. Several previous suicidal attempts.	Verapamil 24,000 mg (as extended release tablets 240 mg) Escitalopram 280 mg	3 days	12 h after intake: Somnolent. BP 80/35 mmHg, HR 35 increasing to 50. Anuria. Muscle twitches and cramps in the extremities.	ABG: pH 7.33, BE −10.8, lactate 1,6–3,2 mmol/l. P-glucose 9.1 mmol/l, P-calcium (ion)1.17 mmol/l Increasing creatinine ECG: Initially AV nodal rhythm, atrial fibrillation with LBBB	Gastric aspiration (without lavage) without any tablets retrieved. Activated charcoal - single dose. Atropine, Isoprenaline, norepinephrine (up to 0.56 μg/kg/minute), epinephrine (up to 0.4 μg/kg/minute). Levosimendan. Other: Plasmapheresis and CRRT, temporary pacemaker (bradycardia), non-invasive ventilation due to carbon dioxide retention
Male, 49 y	Anxiety Periodic Depression, Epilepsy.	Verapamil (as extended release tablets 240 mg) Quetiapine Codeine Acetaminophen Tramadol Topiramate	2 h	Within 12 h after intake: GCS 3. Temperature 32,5 °C. Seizure in ambulance, BP low, cold and pale. Low urine production.	ABG: pH 6.88, lactate 11.3 mmol/L. ECG: ST-depression V2-V6 turning into first bradycardia, then PEA and finally cardiac arrest.	Gastric aspiration (without lavage) with small amounts of tablets retrieved. Activated charcoal - single dose. Endotracheal intubation Atropine, epinephrine, norepinephrine, phenylephrine, Other: Sodium bicarbonate, calcium, dobutamine, naloxone After PEA: Glucagon, amiodarone, infusion with insulin-glucose (high dose).
Female 76 y	Obesity. COPD. Arterial hypertension. Anxiety disorder, Several previous suicidal attempts.	Verapamil Acetaminophen ozaxepam	12 h	Time unknown. Admission status: GCS 9. Temperature 32,7 °C. BP 80/50 mmHg, HR 40 initially increases to 65. AKI. Myoglobin 1167 μg/L.	ABG: pH 7.2, BE 3, lactate 2.4 mmol/L P-glucose 16,1 mmol/L ECG: Bradycardia, 3rd degree AV-block, asystole turning into cardiac arrest.	(GID not performed) Atropine, isoprenaline, norepinephrine (up to 0.7 μg/kilo/minute), epinephrine (0.4 μg/kilo/minute). Other: Flumazenil, N-acetylcysteine, calcium, dopamine, sodium bicarbonate, naloxone.
Female 63 y	Unknown.	Amlodipine 425 mg, Metoprolol 4250 mg Enalapril	2 days	6 h after intake: Comatose. MAP 50, HR 44 (SR), cold. Anuria.	ABG: pH 7.1, BE-19.8, lactate 17 mmol/L. ECG: Atrioventricular Junctional Rhythm (25–40 bpm)	Gastric aspiration (without lavage) with unknown result. Activated charcoal - single dose. Endotracheal intubation, controlled ventilation with high oxygen demand. Atropine, norepinephrine, epinephrine, isoprenaline. Levosimendan, Other: temporary pacemaker, calcium infusion, glucagon, intravenous lipid emulsion. Dialysis.
Female, 61 y	COPD. Depression. Multiple previous suicide attempts.	Verapamil 6000 mg Ramipril 150 mg Ibuprofen 20 g Zolpidem 200 mg	2	Time unknown. Admission status: Unconscious. Reacts to pain. Mumbles. Temperature 34,2 °C. BP 60/ 35, HR 36, cold, pale and sweaty. Anuria.	ABG: pH 7.26, BE − 6,8, lactate 11.1 mmol/L ECG: Wide QRS-complexes.	Activated charcoal - single dose. Endotracheal intubation. Atropine isoprenaline, noradrenaline, adrenaline, dopamine, ephedrine. Other: temporary pacemaker, calcium, glucagon, Calcium (bolus and infusion), glucagon, insulin, flumazenil. Dialysis. After temperature increases to 39 °C: Tazobactam/piperacillin, ciprofloxacin, metronidazole.
Male, 83 y	Paranoid psychosis Cerebral infarction Dementia Arterial hypertension. Aortic valve insufficiency.	Felodipine (as 5 mg tablets) Acetaminophen 10 g Baclofen Quinine 4 g, Acetylsalicylic acid (as 75 mg tablets)	8 days	Time unknown. Admission status: Somnolence. BP 111/45, HR 77 Urine production.	ABG: pH 7.24, BE 8 P-creatinine: 207 mmol/L P-calcium (ion): 0,96 ECG: 1st degree AV block, Right bundle branch block (pre-existing) (	(GID nor performed). adrenalin, noradrenalin Endotracheal intubation. Other: calcium, sodium bicarbonate, N-acetylcysteine. Furosemide, labetalol. Symptomatic treatment (morphine, diazepam)
Male, 30 y	Depression Hypertension	Amlodipine (as 10 mg tablets) Acetylsalicylic acid (as 75 mg tablets) Ramipril	12 h	Time unknown. Admission status: Awake and fully aware of the situation. Nausea and sweaty – otherwise as usual.	P-salicylate 0.4 mmol/L ECG: Normal No further test - patient leaves the hospital.	Activated charcoal - single dose. *Patient leaves the hospital before any other treatment is started. The patient dies 12 h later of carbon monoxide poisoning.*

Abbreviations: *ABG* Arterial Blood Gas, *COPD* Chronic Obstructive Pulmonary Disease, *CRRT* Continous Renal Replacement Therapy, *ECG* ElectroCardioGram, *GCS* Glasgow Coma Score, *BE* Base Excess, *LBBB* Left Bundle Branch Blockade, *GID* Gastrointestinal Decontamination

## Abbreviations

(CCB): Calcium channel blocker
(DDDs): Defined Daily Doses
(DPIC): Danish Poisons Information Center
(ICD-10): International Classification of Diseases 10th Edition
